# SERS and DFT study of copper surfaces coated with corrosion inhibitor

**DOI:** 10.3762/bjnano.5.258

**Published:** 2014-12-29

**Authors:** Maurizio Muniz-Miranda, Francesco Muniz-Miranda, Stefano Caporali

**Affiliations:** 1 Department of Chemistry “Ugo Schiff”, University of Florence, Via Lastruccia 3, 50019 Sesto Fiorentino, Italy; 2 Department of Chemistry and Geology, University of Modena and Reggio Emilia, Via Campi 183, 41125 Modena, Italy

**Keywords:** copper corrosion, DFT, inhibitor film, 1,2,4-triazole, SERS

## Abstract

Azole derivatives are common inhibitors of copper corrosion due to the chemical adsorption occurring on the metal surface that gives rise to a protective film. In particular, 1,2,4-triazole performs comparable to benzotriazole, which is much more widely used, but is by no means an environmentally friendly agent. In this study, we have analyzed the adsorption of 1,2,4-triazole on copper by taking advantage of the surface-enhanced Raman scattering (SERS) effect, which highlights the vibrational features of organic ligand monolayers adhering to rough surfaces of some metals such as gold, silver and copper. To ensure the necessary SERS activation, a roughening procedure was implemented on the copper substrates, resulting in nanoscale surface structures, as evidenced by microscopic investigation. To obtain sufficient information on the molecule–metal interaction and the formation of an anticorrosive thin film, the SERS spectra were interpreted with the aid of theoretical calculations based on the density functional theory (DFT) approach.

## Introduction

Copper has a long history in a variety of industrial uses due to its large electrical and thermal conductivity, mechanical workability and durability (due to its endurance to weathering). These properties, however, can be compromised by the occurrence of corrosion. In fact, copper undergoes severe corrosion in the presence of ions such as chlorides, which can be present in high amount in the environment, aqueous solutions or soil. A very efficient way to protect copper surfaces is by creating an anticorrosive thin film by chemisorption of organic inhibitors. Heterocycles containing sulphur or nitrogen atoms, available for bonding with the copper surface, are widely employed for such a purpose. A class of very efficient corrosion inhibitors for copper and its alloys in different media is formed by 1,2,4-triazole and its derivatives [1–6]. In particular, 1,2,4-triazole exhibits comparable corrosion inhibition [7] as compared to benzotriazole, which is much more widely but is not environmentally friendly.

The adsorption of various organic ligands onto smooth surfaces of copper was previously studied by means of surface-enhanced Raman scattering (SERS) spectroscopy [8]. Due to the huge amplification of the Raman signal of the adsorbed molecules, this technique allows detailed information on the vibrational behavior of molecules adhering to rough surfaces of metals (such as silver, gold, or copper), as well as on the type of interaction with the active sites of the metal substrate to be obtained. In the case of smooth surfaces of copper, however, the SERS activation was ensured by the deposition of silver colloidal nanoparticles on the copper substrate where the organic molecules were already stable and present due to chemisorption [9–11]. Regardless, it must be taken into account that the deposited silver particles, in addition to promoting the SERS enhancement, could to some extent change the ligand adsorption to copper. Hence, a roughening procedure of smooth copper plates was developed to ensure a suitable SERS activation without the aid of silver nanoparticles. The SERS spectra of 1,2,4-triazole adsorbed on nanostructured copper surfaces are interpreted with the aid of density functional theory (DFT) calculations, which were able to provide useful information on the adsorption of different ligands on metal surfaces, including corrosion inhibitors [12–15]. Combining spectroscopic and theoretical results leads to the conclusion that an anticorrosive film coating can be formed by neutral molecules linked to the Cu^+^ active sites of the rough metal surface.

## Results and Discussion

### SEM analysis and profilometry

**Figure 1:**
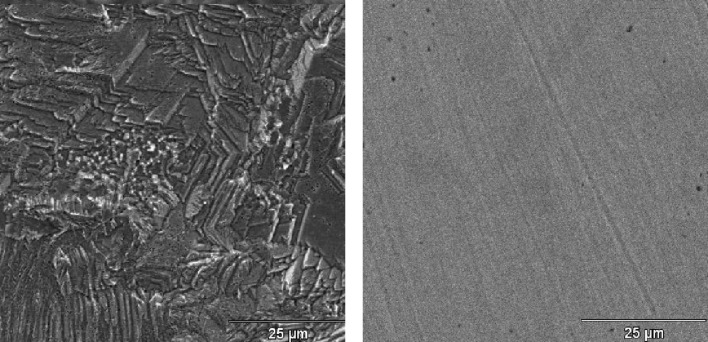
Comparison between the SEM images of an etched copper surface (left) and a smooth copper surface (right).

In order to exploit the Raman enhancement effect of a monolayer of adsorbed molecules on a copper plate, the substrate must exhibit a surface roughness at the nanometer level. In these nanoscale structures, the excitation of electrons from the metal surface by laser irradiation can be confined, resulting in plasmon resonance [16]. The existence of this resonance is a necessary condition to observe a SERS signal by adsorbed species on the metal surface. In order to obtain a suitable surface roughness from a smooth copper substrate, etching in nitric acid was performed (as previously demonstrated [17]), followed by immersion in ammonia solution. The reducing environment limits the oxidation of the copper surface (which takes place very quickly) in order to preserve the metallic nanostructures necessary for the SERS activation by removing oxides by formation of water-soluble complexes with ammonia. Next, the copper plate was immersed in a solution of 1,2,4-triazole, which acts as a corrosion inhibitor by adsorption onto the metallic substrate. The scanning electron microscopy (SEM) images (Figure 1) show that the smooth surface is eroded by the etching treatment to a different extent depending upon the size and orientation of the copper grains in the lamina.

**Figure 2:**
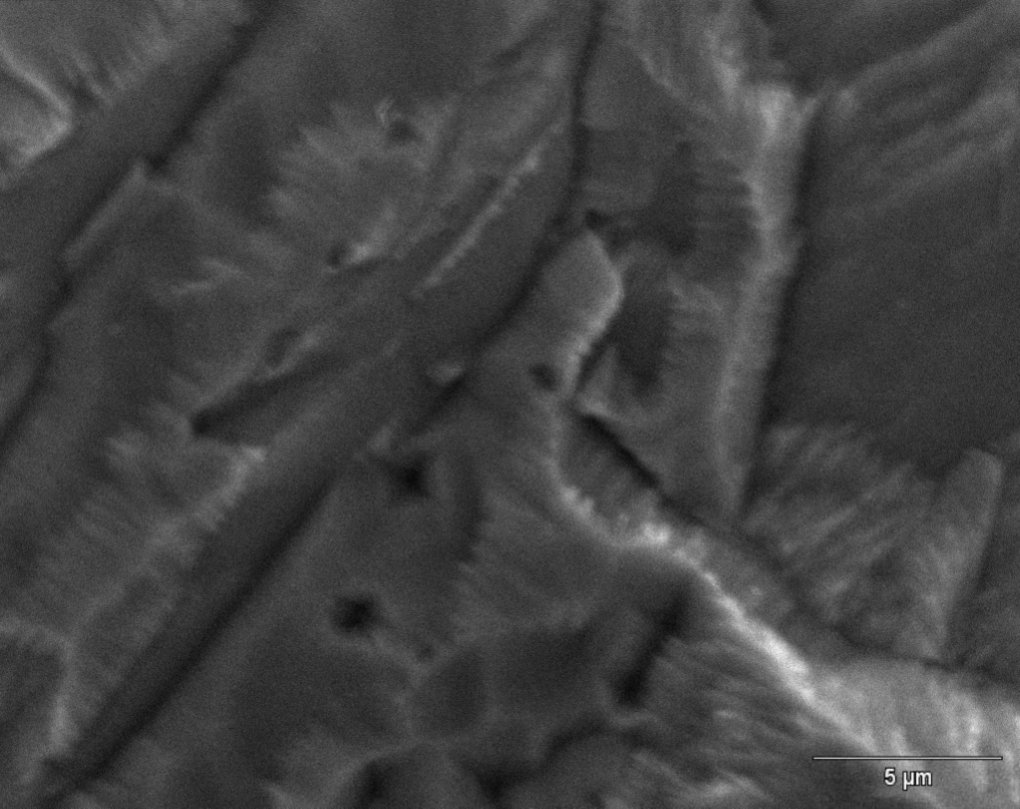
High magnification SEM image of an etched copper surface, showing the fine dendritic structure.

A higher magnification SEM micrograph (Figure 2) of the etched sample surface reveals a fine surface arrangement, showing the formation of submicrometer dendritic structures, typical of rapid growth crystals.

**Figure 3:**
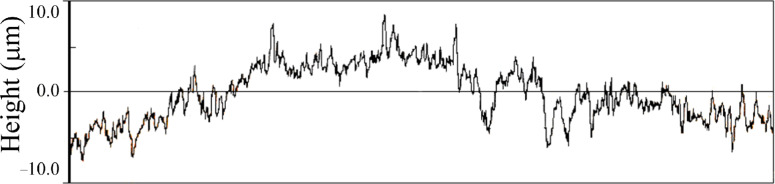
Line profile (profilometry) of an etched copper surface. Total scan length 4.8 mm.

The existence of these nanostructures is additionally proved by measuring the surface roughness. A line profile was obtained on the etched sample surface as shown in Figure 3. In addition to profile variations of a few micrometers due to the copper grains, additional submicrometer variations can be observed and are likely attributable to the dendritic, nanometer-sized structures, which could confer a suitable SERS activation to the metal substrate.

### Raman spectra and DFT analysis

The investigation on the layer of adsorbed ligands is based on the analysis of the SERS spectra, in addition to a computational approach using the DFT method. This study is complicated by the fact that the molecule in question is a heterocyclic ring that can bind to the metal via two different molecular sites, namely, the sp^2^-type nitrogen atoms, N2 and N4, which have electronic lone pairs capable of interacting with the active sites of the copper surface. A further complication arises from the existence of two possible tautomers of 1,2,4-triazole [18–20], which hereafter are denoted as 1*H* and 4*H*, with regard to the presence of a hydrogen atom linked to the nitrogen atom N1 or N4, respectively. In order to interpret the SERS spectra of the adsorbed molecules, it is first necessary to perform a preliminary vibrational analysis of the isolated molecule.

**Figure 4:**
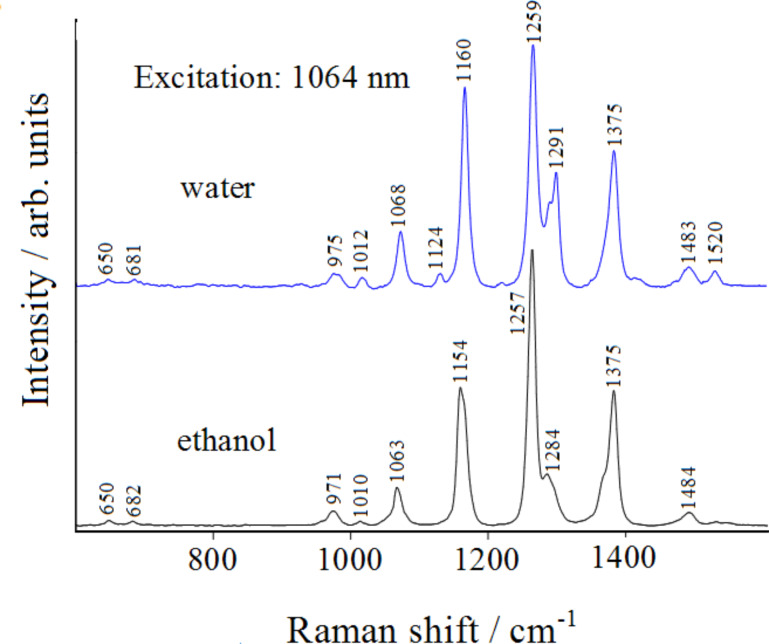
Normal FT-Raman spectra of 1,2,4-triazole in solution. Solvent subtracted.

Figure 4 shows a typical FT-Raman spectra of 1,2,4-triazole in ethanol and water solutions. They are quite similar except for the band observed in water at 1291 cm^−1^ and in ethanol at 1284 cm^−1^. These observed frequencies can be calculated for the isolated molecule by DFT.

The DFT calculations show that the 1*H* tautomer is more stable than the 4*H* having an energy of −242.320444 Hartree and −242.309585 Hartree, respectively, as discussed in the literature [18]. In addition, the calculated free energy difference indicates that the 1*H* tautomer is more stable than 4*H*, resulting in ΔG = 6.22 kcal/mol, the same value found by Jimenez and Alderete [21] using the same theory level (B3LYP/6-311++G(d,p)) with a different computational package. Table 1 shows the observed Raman frequencies of the 1,2,4-triazole solution, as compared with those calculated for the 1*H* and 4*H* tautomers, along with an approximate assignment.

**Table 1:** Observed and calculated Raman shifts (cm^−1^) of 1,2,4-triazole.

Obs. Raman water	Obs. Raman ethanol	Calc. 1*H* tautomer	Calc. 4*H* tautomer	Description^a^

		541	509	o.p. H bending
650 vw	650 vw	663	644	ring torsion
681 vw	682 vw	682	672	ring torsion
			777	
		827	814	o.p. H bending
		878		o.p. H bending
		943	922	ring bending
975 w	971 w	980	951	ring bending
1012 w	1010 vw		999	
1068 m	1063 m	1056	1071	N1-N2 stretching
1124 w		1115	1087	i.p. H bending
1160 s	1154 m/s	1156		N1–N2, C3–N4 stretching
1213 vw			1204	
1259 vvs	1257 vvs	1250	1278	ring breathing
1291 m	1284 sh	1291		N4–C3 stretching, i.p. H bending
1375 m/s	1375 m/s	1359	1377	N2–C3, N4–C5 stretching
1414 vw		1432		i.p. H bending
1483 w	1484 w		1488	
			1490	
1520 w	1522 vw	1520		N2–C3 stretching

^a^For 1*H* tautomer (see Figure 5); o.p. = out-of-plane; i.p. = in-plane.

From the inspection of Table 1 one can see that the calculated frequencies of the 1*H* tautomer agree well with the intense Raman bands observed in solution. The weak Raman band at 1480 cm^−1^ can be attributed to the 4*H* tautomer which is present in a small amount.

**Figure 5:**
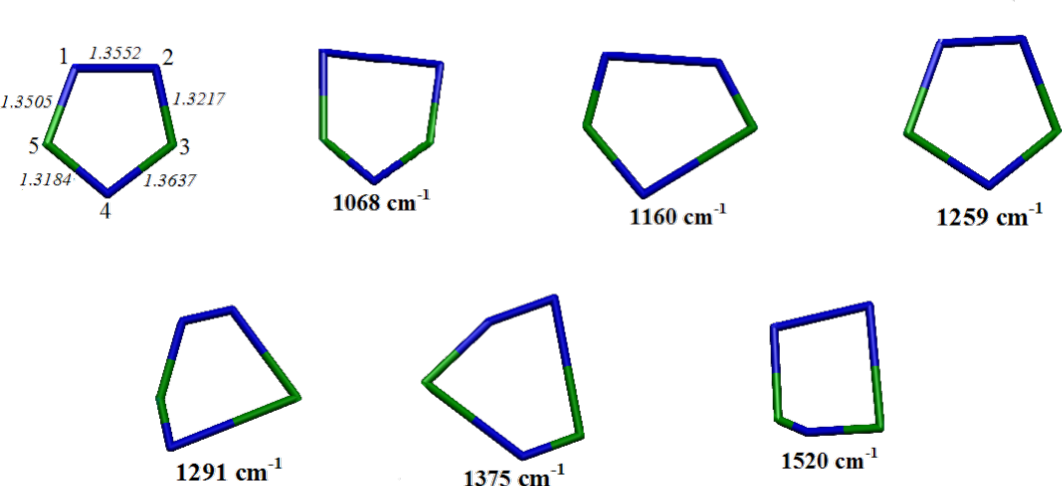
Optimized geometry of 1*H*-1,2,4-triazole (upper left) with the calculated distances (angstroms), along with the vibrational modes corresponding to the strongest Raman bands. Hydrogen atoms are hidden.

The optimized geometry of the 1*H* tautomer, along with the vibrational modes corresponding to the strongest Raman bands, are shown in Figure 5.

The N2–C3 and N4–C5 bonds are the shortest double bonds according to the experimental structure [22–23]. In general, the calculated distances are very similar to the experimental values, which have been reproduced here better than with previous computational approaches [12,24–25].

**Figure 6:**
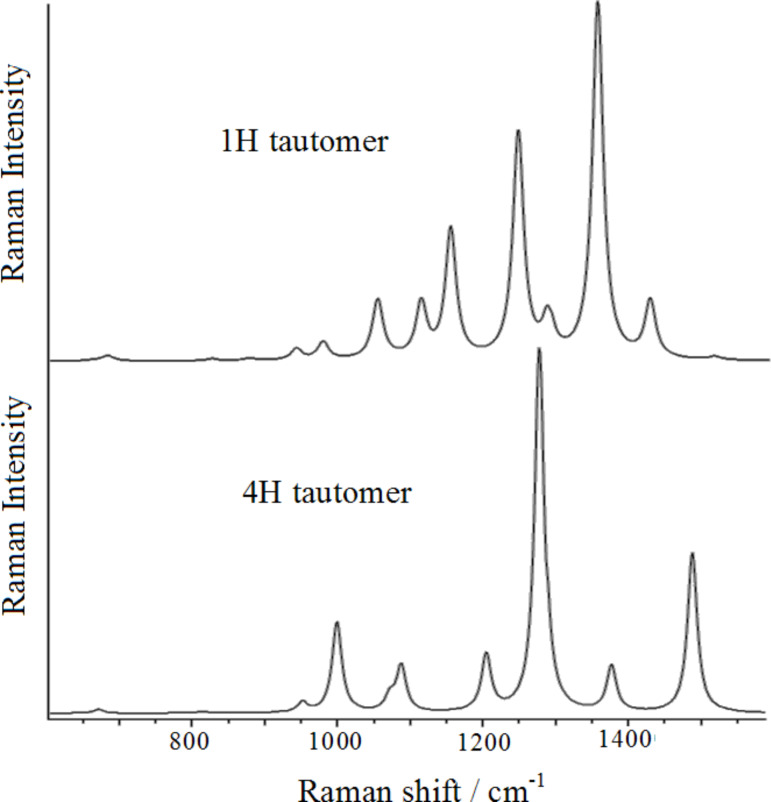
Simulated Raman spectra of the two tautomers of 1,2,4-triazole.

The vibrational normal modes shown in Figure 5, which correspond to the most intense Raman bands, are all ring deformations: the Raman bands at 1068, 1291 and 1520 cm^−1^ are mainly attributable to N1–N2, N4–C3 and N2–C3 stretching modes, respectively, whereas those observed at 1160, 1259 and 1375 cm^−1^ can be described as N1–N2/C3–N4 stretching mode, ring breathing mode and N2–C3/N4–C5 stretching mode, respectively. It should be noted that the modes that involve the stretching of two bonds, namely those at 1160 and 1375 cm^−1^, along with the ring breathing mode at 1259 cm^−1^, result in a larger increase of the ring size and consequently of the molecular polarizability, resulting stronger Raman intensities, as well as was experimentally observed. In Figure 6, we show the calculated Raman spectra of the two tautomers with their relative intensities. The observed Raman spectrum of triazole is better reproduced as compared to that simulated for the 1*H* tautomer, where only the band around 1380 cm^−1^ appears overestimated.

### SERS investigation

**Figure 7:**
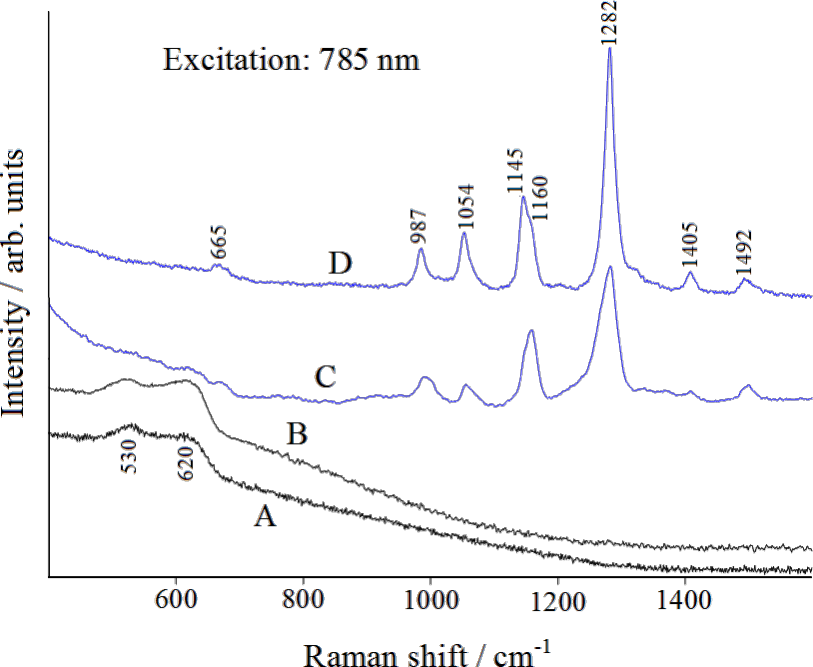
Micro-Raman spectra (excitation 785 nm): smooth copper plate (A); etched copper plate (B); etched copper plate, after immersion in an ethanol (C) or a water (D) solution of 1,2,4-triazole and air-drying.

On the basis of these structural and vibrational results on the isolated molecule, we can assume that the DFT calculations (at the B3LYP/6-311++G(d,p) level of theory, as described in the Computational details section) are reliable, such that they can be applied to the models of triazole/copper complexes. However, a prediction regarding the molecular sites of interaction with the copper surface can already be made. In fact, in the 1*H* tautomer, the nitrogen atom N4 is more negatively charged than N2, based on evaluation of the Mulliken atomic charges (−0.2059∙|*e|* for N4, −0.1450∙|*e|* for N2, where |*e|* is the elementary electric charge). Consequently, it is expected to be more favorably linked to metal. The 4*H* tautomer, instead, has the opportunity to interact with one or two atoms of copper. Figure 7 shows the Raman spectra recorded on the copper plates upon excitation at 785 nm.

In this figure, (A) is the spectrum of the smooth plate of copper exposed to air, (B) is that of the plate exposed to air after the etching treatment, before immersion in a triazole solution. In both cases, only two broad Raman bands occur around 530 and 620 cm^−1^, which are related to the presence of Cu(I) oxide, as reported in the literature [26–28]. These bands, visible on both the smooth and etched copper surfaces, can be related to the formation of a Cu_2_O multilayer. On the contrary, the oxidation reaction of the etched copper surface is not evidenced when adsorbed triazole is present. Spectrum C in Figure 7 corresponds to the etched copper plate after immersion in an ethanol solution of triazole and air-drying: the Raman bands attributable to the presence of adsorbed triazole are observed, with sizeable frequency shifts with respect to the corresponding normal Raman bands of triazole in solution (see Table 1). This indicates that the etching treatment provides the necessary SERS activation of the copper surface and that triazole is chemisorbed on the copper surface. Without this surface treatment, no Raman signal from the adsorbed ligand could be detected. The same SERS bands are also observed with larger intensities for the etched plate immersed in an aqueous solution of triazole (spectra D). Again, in this case, the spectrum does not show Raman bands due to the formation of cuprous oxide, which is related to the initial stage of the corrosion process of the metal. Therefore, the SERS findings highlight the corrosion inhibition effect by the layer of adsorbed triazole.

**Figure 8:**
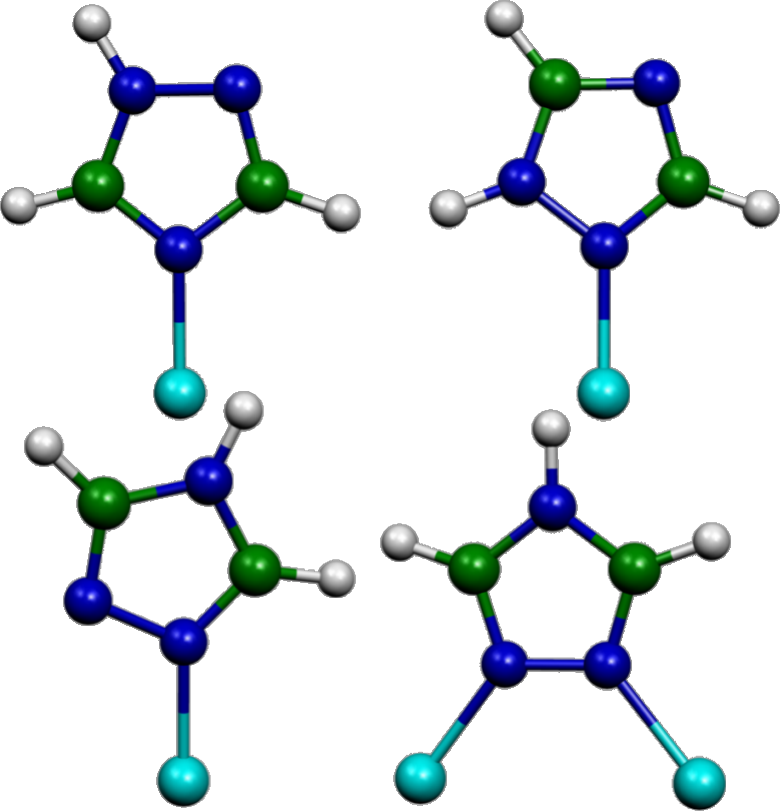
Model systems for 1*H*/copper (upper panel) and 4*H*/copper (lower panel) complexes; carbon, nitrogen and copper atoms are represented as green, dark blue and light blue spheres, respectively.

To understand this effect and to obtain information on the inhibiting layer, it is possible to obtain additional information from the SERS data using the DFT computational approach. This approach is based on model systems formed by the ligand molecules and the active sites of the copper surface (see Figure 8), which can be formed by Cu^0^ neutral atoms or Cu^+^ cations.

Here, we have considered the complexes of the 1*H* tautomer interacting through the N2 or N4 atom (Figure 8, upper panel) with a Cu^+^ ion or with a Cu^0^ neutral atom. In addition, the complexes of the 4*H* tautomer interacting with a Cu^+^ ion or with a Cu^0^ neutral atom have been taken into account, as well as with two Cu^+^ ions or with a Cu^+^/Cu^0^ couple (Figure 8, lower panel). The structure of the 4*H* complex with two neutral copper atoms did not converge and even adopted a more relaxed configuration for the structural optimization.

Table 2 shows the comparison between the observed SERS bands and those calculated for the different model systems by DFT calculations. Only the complex formed by the 1*H* tautomer bound to a Cu^+^ ion through the N4 atom provided calculated frequencies that reasonably agree with those observed in the SERS spectra. Moreover, even the calculations on the isolated molecule led favored the 1*H* tautomer interacting with the N4 atom rather than with the N2 atom. The interaction with Cu^+^ ions is indeed possible, considering the oxidation facility of the copper surface; moreover, in the DFT calculations, the interaction of ligand molecules with Cu^+^ ions always leads to the formation of complexes in singlet electronic states (spin multiplicity 1). These are more stable than those with neutral copper atoms, with spin multiplicity 2. Indeed, it should be noted that the interaction with a Cu^+^ ion provides a stronger electronic charge-transfer from molecule to metal with respect to the interaction with a neutral copper atom, about 0.5∙|*e|* versus 0.3∙|*e|*.

**Table 2:** Observed SERS wavenumbers (cm^−1^) of adsorbed 1,2,4-triazole compared with the calculated Raman wavenumbers of the surface complexes^a^.

Obs. SERS on Cu	Calc. 1*H* Cu^+^(4)	Calc. 1*H* Cu^+^(2)	Calc. 4*H* Cu^+^	Calc. 4*H* 2Cu^+^	Calc. 4*H* Cu^+^/Cu^0^	Calc. 4*H* Cu^0^	Calc. 1*H* Cu^0^(4)	Calc. 1*H* Cu^0^(2)

		514				545	570	545
	626	639	609	618	608	641	659	656
665 w	665	655	667	661	669	669	673	668
	701		674	721	671			
	851	877	839	875	844	798	838	840
	869	895	861	895	867	828	878	882
	950	932	950	940	951	942	958	944
987 m	1002	1007	958	1002	968	951	974	984
						1014		
1054 m	1059	1088	1043	1051	1023	1079	1052	1066
			1087	1136	1108		1133	1118
1145 s	1137	1122	1137	1148	1118	1100	1145	1168
1160 s	1167	1194						
			1213	1228	1215	1205		
1282 vs	1265	1252	1302			1288	1253	1247
1282 vs	1275	1319		1306	1310		1289	1304
1405 w	1393	1351	1397	1410	1401	1391	1370	1356
								
1492 w	1483	1458	1503	1515	1514	1492	1452	1441
								
	1538	1511	1530	1541	1523	1504	1528	1513

^a^1*H* Cu^+^(4): 1*H* tautomer linked to Cu^+^ via N4; 1*H* Cu^+^(2): 1*H* tautomer linked to Cu^+^ via N2; 1*H* Cu^0^(4): 1*H* tautomer linked to Cu^0^ via N4; 1*H* Cu^0^(2): 1*H* tautomer linked to Cu^0^ via N2; 4*H* Cu^+^: 4*H* tautomer linked to Cu^+^; 4*H* Cu^+^: 4*H* tautomer linked to two Cu^+^; 4*H* Cu^+^/Cu^0^: 4*H* tautomer linked to Cu^+^ and Cu^0^; 4*H* Cu^0^: 4*H* tautomer linked to Cu^0^.

### Corrosion inhibition effect

**Figure 9:**
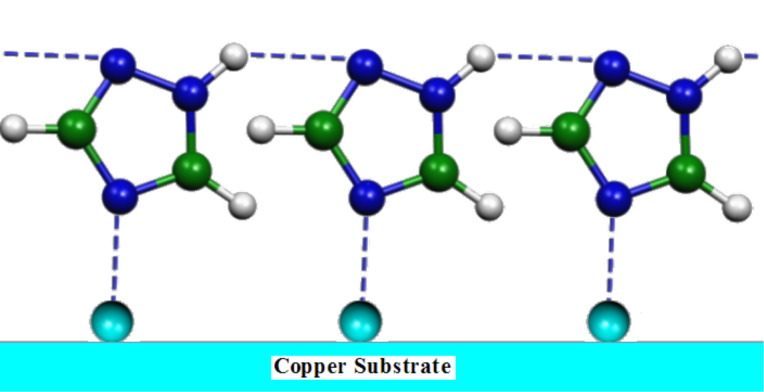
Adsorption model of the 1,2,4-triazole molecules on the copper substrate.

Considering the interaction of the molecule with the metal surface via the N4 atom, the possibility to form chains of adsorbed molecules linked by hydrogen bonds exists, as proposed in Figure 9. This creates a compact, adsorbed triazole layer which can impair surface corrosion and explains the absence of the Raman bands at 530 and 620 cm^−1^ in the SERS spectrum, which would be due to the formation of a Cu(I) oxide multilayer (indicating the initial stage of the corrosion process of the metal).

**Figure 10:**
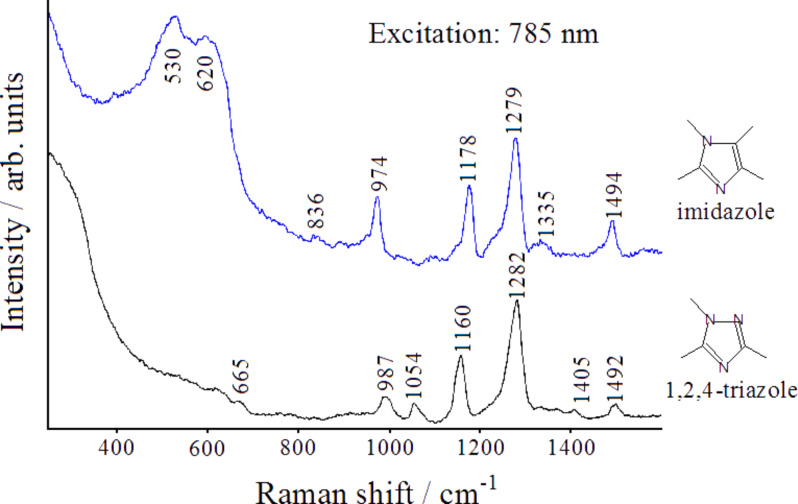
Comparison between the SERS spectra of 1,2,4-triazole and imidazole absorbed on etched copper surfaces, after immersion in ethanol solutions and air-drying.

As a further confirmation of this surface arrangement of adsorbed triazole molecules acting as protective film for copper, we report in Figure 10 the SERS spectrum of another azole, imidazole (structurally quite similar to triazole), obtained by following the same experimental procedures adopted for triazole. The SERS spectra of the two azoles appear similar, which is reasonable considering the same adsorption geometry on copper, however, the SERS spectrum of imidazole shows the occurrence of strong Raman bands around 530 and 620 cm^−1^. These are attributed to a multilayer of cuprous oxide, which are absent in the SERS spectra of triazole. Hence, the adsorbed imidazole molecules, which are unable to interact by hydrogen bonding, do not allow for the excellent inhibition action provided by the compact thin film of adsorbed molecules of triazole, which is capable of isolating the metallic surface from the oxidative action of the atmosphere.

## Conclusion

An etching process was performed on smooth copper surfaces using nitric acid followed by immersion in an ammonia solution, resulting in SERS-active substrates. This activation was validated by the SEM and profilometry investigations of the roughened surface, which show submicrometer structures after etching. The presence of absorbed 1,2,4-triazole molecules is highlighted by the examination of the SERS spectra, obtained by simple incubation of the copper plate in ligand solutions and air-drying. This treatment inhibits the oxidation of the copper surface, as was evidenced by the absence of typical bands of Cu(I) oxide in the Raman spectra. Additionally, the observed frequency shifts of the SERS spectral peaks with respect to the corresponding non-SERS Raman spectral peaks of triazole in solution suggest chemisorption of the ligand molecules on the copper surface. The DFT calculations on molecule/metal model systems assisted in the interpretation of the spectroscopic data and provided information on both the tautomeric form of 1,2,4-triazole adsorbed on copper, and the nature of the surface active sites interacting with the ligand molecules. A model of adsorption based on a compact molecular film was proposed, thus justifying the inhibiting action of 1,2,4-triazole with respect to the corrosion of the copper surface. The SERS study of imidazole, similar to triazole but unable to bind the adsorbed molecules among them, confirms this conclusion.

## Experimental

A hot-rolled plate of copper, as supplied by Aldrich (purity 99.98%), was first mechanically polished with alumina powder to a mirror finish then carefully washed with water and ethanol in an ultrasonic bath. The smooth plate was immersed for one minute in a concentrated solution of nitric acid in order to obtain a discernable etching and then the plate was immersed in a concentrated solution of ammonia to eliminate the presence of copper oxides from the surface. After a quick washing with water and ethanol as running solvents, the etched plate was immersed for one day in a diluted solution (10^−2^ M) of 1,2,4-triazole, then carefully washed with ethanol to leave behind only the chemisorbed ligand. Sample morphology was observed by SEM (Hitachi S-2300) operating at 20 kV. The surface roughness was measured by collecting line profiles using a Hommel Tester W55 profilometer, scanning 4.8 mm at a 0.2 mm s^−1^ scan rate. The parameters employed were λ_c_ = 0.8 mm and λ_c_/λ_s_ = 300 using a filter ISO 11562(MI).

Raman spectra of 1,2,4-triazole (10^−1^ M concentration) in ethanol or water solutions were collected with a Fourier transform (FT)-Raman spectrometer (Bruker Optics, Model MultiRam), equipped with a broad range quartz beamsplitter, an air-cooled Nd:YAG laser excitation source (1064 nm) and a Ge diode detector cooled with liquid nitrogen. The instrument provided a spectral range of 3600–50 cm^−1^ (Stokes shift). The experiments were performed in a 180° geometry, with 200 mW of laser power.

Raman spectra on copper plates were measured using a Renishaw RM2000 microRaman apparatus, equipped with a diode laser emitting at 785 nm. Sample irradiation was accomplished by using the 50× microscope objective of a Leica Microscope DMLM. The backscattered Raman signal was filtered by a double holographic notch filter system and detected by an air-cooled CCD. All spectra were calibrated with respect to a silicon wafer at 520 cm^−1^.

### Computational details

All calculations were carried out using the Gaussian 09 package [29]. Optimized geometries, vibrational frequencies and other molecular properties of 1,2,4-triazole and its investigated copper complexes were obtained using the hybrid B3LYP exchange-correlation function [30–33]. The 6-311++G(d,p) basis set was used for all atoms, including copper. The integral grid was set to “ultrafine” and the optimization criteria to “very tight”. By allowing all the parameters to relax, the calculations converged to optimized geometries corresponding to true energy minima, as revealed by the lack of imaginary values in the vibrational mode calculations. A scaling factor of 0.98 for all calculated vibrational wavenumbers was adopted, as performed for similar molecular systems [13,15,34–36].
